# 

**Published:** 1911-09-30

**Authors:** 


### THE GLASGOW EXTENSIONS.

We understand that the opening of the clinical laboratory and of the new wards, now all but finished, at the Western Infirmary, Glasgow, has been fixed for Friday, October 13, and not the 6th inst., as previously stated.

### APPOINTMENTS VACANT.

Full particulars of these vacancies can be obtained on reference to our advertisement columns :

Addenbrooke Hospital, Cambridge.—Second House Surgeon.
Bradford Royal Infirmary.—House Physician.
City of Birmingham Education Committee.—Medical Officer.
Cornwall County Asylum, Bodmin.—Third Assistant Medical Officer.
Croydon General Hospital.—Junior House Surgeon.
Leeds General Infirmary.—Resident Medical Officer.
Royal Free Hospital.—House Physician and House Surgeon (male).
Salford Royal Hospital.—Casualty House Surgeon.
Seamen's Hospital Society, Greenwich.—Two House Physicians, two House Surgeons, and Honorary Anæsthetist.
Winnipeg General Hospital.—Superintendent.

### GIFTS.

The Bradford Royal Infirmary and the Bradford Children's Hospital both receive legacies of £500 from the estate of the late Sir Theo. Peel, of Park Gate, Guiseley.

Mr. Daniel Layborn, of Radnor and West Liverpool, has left, subject to certain life interests, about £10,000 equally between the Children's Infirmary, Myrtle Street, Liverpool; the Liverpool Bluecoat Hospital; the Beverley Cottage Hospital; the Queen Victoria District Nursing Association, Liverpool; and the Liverpool Children's Friend Society.

The Gravesend District President of the League of Mercy, Col. Sir Fitzroy D. Maclean, Bart., K.C.B., has notified the Hon. Treasurer of the local hospital that the Distribution Committee have granted £30 towards its funds.

Mr. Edmund Birch Gibson, of Saffron Walden, has left £500 to the Saffron Walden Hospital.

Mr. Alexander Hogg Nasmyth, of Dunfermline, Fife, has left £500 each to the Royal Edinburgh Hospital for Sick Children, the Longmore Hospital for Incurables at Edinburgh, and to the Royal Blind Asylum of that city.

Alderman Henry Goody, of Colchester, has left £100 to the Essex County Hospital, Colchester.

Under the will of Mrs. Jane Wright, of Potton, Beds, the County Hospital, Bedford, and Addenbrooke's Hospital, Cambridge, will receive a certain proportion of the residue of the estate, probably amounting to about £1,200 each.

### BUILDINGS CONTEMPLATED AND IN PROGRESS.

General Infirmary, Worcester.—It is announced that the Infirmary Committee has accepted the tender of Messrs. Joseph Wood and Sons, Worcester, to build the City of Worcester's King Edward VII. Memorial Annexe to the Infirmary. This firm built the Nurses' Home, which was opened in the year 1898.

